# Identification of MOR-Positive B Cell as Possible Innovative Biomarker (Mu Lympho-Marker) for Chronic Pain Diagnosis in Patients with Fibromyalgia and Osteoarthritis Diseases †

**DOI:** 10.3390/ijms21041499

**Published:** 2020-02-22

**Authors:** William Raffaeli, Valentina Malafoglia, Antonello Bonci, Michael Tenti, Sara Ilari, Paola Gremigni, Cristina Iannuccelli, Chiara Gioia, Manuela Di Franco, Vincenzo Mollace, Laura Vitiello, Carlo Tomino, Carolina Muscoli

**Affiliations:** 1 ISAL Foundation, Institute for Research on Pain, 47922 Torre Pedrera, Italy; antonello.bonci@yahoo.com (A.B.); tenti1990@gmail.com (M.T.); 2 Global Institute on Addictions, 1501 Biscayne Blvd, FL 33132, USA; 3 Institute of Research for Food Safety & Health (IRC_FSH), Department of Health Sciences, University ‘Magna Graecia’ of Catanzaro, 88100 Catanzaro, Italy; sara.ilari@hotmail.it (S.I.); mollace@unicz.it (V.M.); muscoli@unicz.it (C.M.); 4 Department of Psychology, University of Bologna, 40127 Bologna, Italy; paola.gremigni2@unibo.it; 5 Rheumatology Unit, Department of Internal Medicine and Medical Specialities, Sapienza University of Rome, 00161 Rome, Italy; cristina.iannuccelli@uniroma1.it (C.I.); gochiaragioia@gmail.com (C.G.); manuela.difranco@uniroma1.it (M.D.F.); 6 Unit of Flow Cytometry, IRCCS San Raffaele Pisana, 00166 Rome, Italy; laura.vitiello@sanraffaele.it; 7 Scientific Direction, IRCCS San Raffaele Pisana, 00166 Rome, Italy; carlo.tomino@sanraffaele.it

**Keywords:** chronic pain dDiagnosis, chronic pain biomarkers, opioid receptors, lymphocytes, fibromyalgia, osteoarthritis

## Abstract

Fibromyalgia (FM) diagnosis follows the American College of Rheumatology (ACR) criteria, based on clinical evaluation and written questionnaires without any objective diagnostic tool. The lack of specific biomarkers is a tragic aspect for FM and chronic pain diseases in general. Interestingly, the endogenous opioid system is close to the immune one because of the expression of opioid receptors on lymphocytes membrane. Here we analyzed the role of the Mu opioid receptor on B lymphocytes as a specific biomarker for FM and osteoarthritis (OA) patients. We enrolled three groups of females: FM patients, OA patients (chronic pain control group) and healthy subjects (pain-free negative control group). We collected blood samples to apply immunophenotyping analysis. Written tests were administrated for psychological analysis. Data were statistically analyzed. Final results showed that the percentage of Mu-positive B cells were statistically lower in FM and OA patients than in pain-free subjects. A low expression of Mu-positive B cell was not associated with the psychological characteristics investigated. In conclusion, here we propose the percentage of Mu-positive B cells as a biological marker for an objective diagnosis of chronic pain suffering patients, also contributing to the legitimacy of FM as a truly painful disease.

## 1. Introduction

Fibromyalgia (FM) is one of the most disabling chronic pain syndromes [1] affecting especially women and middle-aged people with a global prevalence between 0.2% and 6.6% [2,3,4]. In addition to chronic pain, hallmark symptoms of FM often include chronic fatigue, sleep disturbances, cognitive dysfunctions (the so-called “fibro-fog”) and other somatic symptoms [5].Moreover, from a psychological viewpoint, FM is associated with distress in terms of depression, anxiety and stress-related symptoms [1,6]. Within the last International Association for the Study of Pain (IASP) meeting, FM has been included in the International Classification of Diseases (ICD-11) inside the category of “chronic primary pain” [7]. Thus, FM is now recognized by specialists as a pathological condition, characterized by a chronic primary pain state, which could be assimilated as a disease in its own right, as already proposed by Raffaeli and colleagues [8]. However, FM is sometimes still considered as a psychological illness and not a “real disease”, and FM patients are frequently labeled with the diagnoses of “somatoform pain disorder” or “affective disorder” [9]. Thus, FM still represents a clinical enigma with real diagnostic difficulties since its pathophysiology is not well known and its symptoms are nonspecific and overlap with many other disorders [10].

The American College of Rheumatology (ACR) criteria for FM diagnosis have changed over time [5,11,12,13], maintaining advantages and limitations [14], and clinicians rely only on clinical examination and questionnaire administration to make a diagnosis [15]. Neither specific diagnostic laboratory tests nor biomarkers are available to confirm FM diagnosis, especially in its specificity of chronic widespread pain (CWP), excluding some groundbreaking tests available in the near future [16]. In this context, delays and misdiagnosis may frequently occur, with relevant consequences on patients’ life and treatment efficacy, as well as socioeconomic burdens on patients and the health care system [17,18,19,20,21]. For these reasons, the identification of objective and specific FM biomarkers is a priority for correct diagnosis. In this field, it has been suggested that FM cannot be diagnosed with a single biomarker, although proteins found to be involved in FM pathogenesis could play an important role in a specific subset of the syndrome [22,23].

Evaluation of cytokines and chemokines levels in stimulated peripheral blood mononuclear cell (PBMC) has shown patterns uniquely present in patients with FM [24,25]. Metabolomics analysis through the innovative technique called “vibrational spectroscopy” [16] has detected a “molecular fingerprint” in blood samples, specific for FM patients. More recently, Niculescu and colleagues [26] have proposed putative “risk genes”, showing a higher expression, and putative “protective/resilience genes”, with a lower expression, in severe pain states. In recent years, attention has been focused on the close association between peripheral nerve and opioid-containing immune cells [27]. Both immune cells and neurons share common ligands and receptors, and this ligand-receptor communication influences and activates cellular pathways in both systems [28]. Immune cells express a myriad of opioid receptors: μ (Mu[MOR]/Oprm1), δ (delta [DOR]/Oprd1) and κ (kappa [KOR]/Oprk1) [29]. Human studies have demonstrated that short-term morphine induces T-lymphocytes cytokines release from T cells, by enhancing the differentiation of B-lymphocytes [30]. Moreover, Mu agonists increase IgM and IgG production by B cells but the ability to produce antibodies also requires the cooperation of other immune cells, expressing opioid receptors, as well. Mu activation by morphine has also been shown to regulate macrophage functions, including nitric oxide production and phagocytosis [31], and macrophages Mu is up-regulated by several cytokines [32]. However, little is known about the role of opioid receptors on circulating cells during the development of a chronic pain disease.

Since the 1990s, Raffaeli and colleagues have investigated the clinical combined role of opioids and the immune system, in order to personalize pain therapy [33,34,35,36,37,38,39], by underlining that clinical use of opioids could interact with the endogenous antinociceptive systems [40] and these interactions are different for diverse pain pathologies [41]. The complex communication between endogenous antinociceptive systems, pathology and peripheral opioid receptors has encouraged us to postulate a role for the lymphocyte opioid Mu receptor as a biomarker for FM diagnosis.

Thus, here we propose an observational, cross-sectional, single blind, diagnostic trial in order to investigate whether Mu opioid receptor on lymphocyte membranes could be considered as a FM biomarker or whether it could be a common marker for different chronic pain syndromes. Moreover, given the high prevalence of psychological distress and psychiatric disorders in FM and more in general in chronic pain [6,42,43], and the identification of different psychobiologic FM profiles [44], we also investigate whether differences in Mu opioid receptor expression could be influenced by psychological aspects relevant in chronic pain diseases and FM. In particular we focused on illness perception, coping and catastrophizing, pain acceptance and depression, as well as anxiety and stress-related symptoms [6,45,46,47,48,49].

## 2. Results

### 2.1. Demographic and Clinical Characteristics 

Following inclusion and exclusion criteria assessment (see 4.2 paragraph), clinical data were collected in a specific Case Report Form (CRF)including: demographics, clinical characteristics and pharmacological history (Table 1).Pain intensity analysis was measured using the 11-point numerical rating scale (NRS) test, where 0= No pain and 10= worst possible pain. The totality of enrolled patients was female (*n* = 102), mean age 51 years (Table 1). Fifty-eight percent (58%) were fibromyalgic patients, 19% were osteoarthritic (OA) patients and the remaining 23% were pain-free people. All of the FM patients (*n* = 59) ranked between moderate (NRS: (4–6); 15.3%) and severe (NRS:(7–10); 84.75%) NRS score. Fifty-nine percent were patients suffering for less than five years, 41% for more than five years. OA patients (*n* = 19) rated with mild pain (NRS: (0–3); 36.87%), moderate (26.4%) and severe pain (36.87%); 63.1% of OA patients had pain for less than five years and the remaining 36.93% for more than five years (Table 2). All patients were following a personal pharmacological therapy prior to enrollment: 41% antidepressants; 14% benzodiazepines; 5% non-steroidal anti-inflammatory drugs (NSAIDs); 19% a combination of the previous drugs; 21% nutritional supplement or nothing. All of the enrolled FM and OA patients undergoing biological investigation received psychological questionnaires and 63 of them (FM patients *n* = 53, OA patients *n* = 10) filled them.

### 2.2. Biological Results

#### 2.2.1. Mu Opioid Receptors on T and B Lymphocytes Membrane

We analyzed the blood samples of all enrolled patients in order to detect the expression of Mu receptors on the membrane of T and B lymphocytes. We used one-way Analysis of Variance (ANOVA) both for group comparisons and for intra-group homogeneity assessment. All data were expressed as mean ±S.E.M. Tukey’s least significant difference multiple comparison was used for post-hoc analysis following one-way ANOVA, to compute the probability values (P) in three-group comparison. A P threshold of 0.05 was considered for statistical significance. 

**Figure 1:**
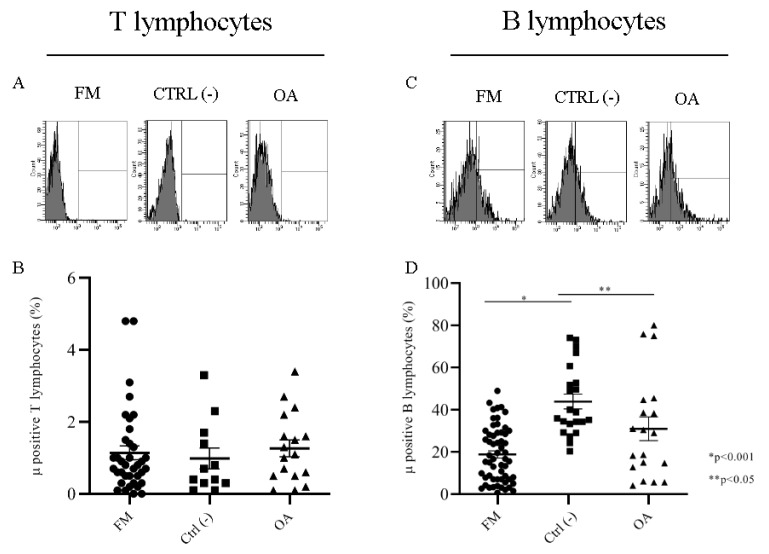
Flow cytometry analysis of the Mu opioid receptor in fibromyalgia (FM), osteoarthritis (OA) and CTRL(−) patients in T lymphocytes (**A**) and B lymphocytes(**C**). Mu+ T cells percentage (**B**) and Mu+ B cells percentage (**D**) in FM, OA, CTRL(−) patient groups.

We found that only a very low (mean < 1.3 ± 0.3) percentage of T lymphocytes from FM and OA patients and CTRL expressed Mu opioid receptor (Figure 1B), and we did not find any significant difference of expression for the Mu receptor between groups. Interestingly, a consistent percentage of B lymphocytes of FM, OA and negative control group patients expressed MOR: we found intra-group homogeneity (FM mean: 18.2 ± 1.7; OA mean: 30.96 ± 5.5; CTRL(−) mean: 43.80 ± 3.5) and significant differences of Mu expression between CTRL and FM (*p* < 0.001), and CTRL and OA ( *p*< 0.05) (Figure 1D).

#### 2.2.2. Correlation between Intensity of Pain (NRS Scale) and Mu-Positive B Cells 

**Figure 2:**
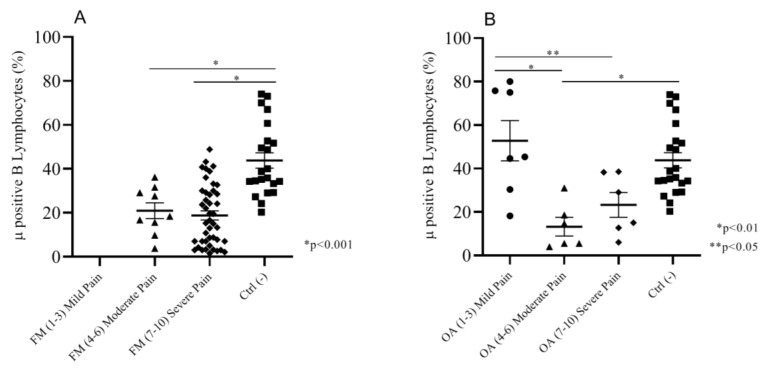
(**A**) Moderate and severe pain (NRS) FM patients showed a significantly lower % of Mu+ B cells than the negative control group patients. (**B**) Moderate and severe pain OA patients showed a significantly lower Mu+ B cell percentage than the mild pain OA and negative control group patients; mild pain OA patients and control group did not show any significant differences in the percentage of Mu+ B cells.

Both FM and OA patients are characterized by pain. Considering that not all of the patients felt pain the same way, we wanted to investigate whether such differences could depend on opioid receptor expression on circulating cells. Thus, we compared MOR expression on B lymphocytes, by focusing on the intensity of pain between FM and OA patients versus the pain-free negative control group. FM patients, ranking between moderate (NRS (4–6)) and severe (NRS (7–10)) pain, did not present any differences in the percentage of Mu-positive (Mu+) B cellsbut both of the subgroups showed a significantly (*p* < 0.001) lower expression of Mu+ B cells (respectively, mean: 20.98 ± 3.5; 18.82 ± 2.0) than the control group (mean: 43.80 ± 3.5) (Figure 2A). OA patients declaring moderate pain (NRS (4–6)) (mean: 13.22 ± 4.2) and severe pain (NRS (7–10)) (mean: 23.25 ± 5.6) showed insignificant differences; mild pain OA patients (NRS (1–3)) presented a significantly higher percentage of Mu+ B cells (mean: 52.77 ± 9.2) but not significantly different from Mu expression of control group patients (mean: 43.80 ± 3.5) (Figure 2B).

**Figure 3:**
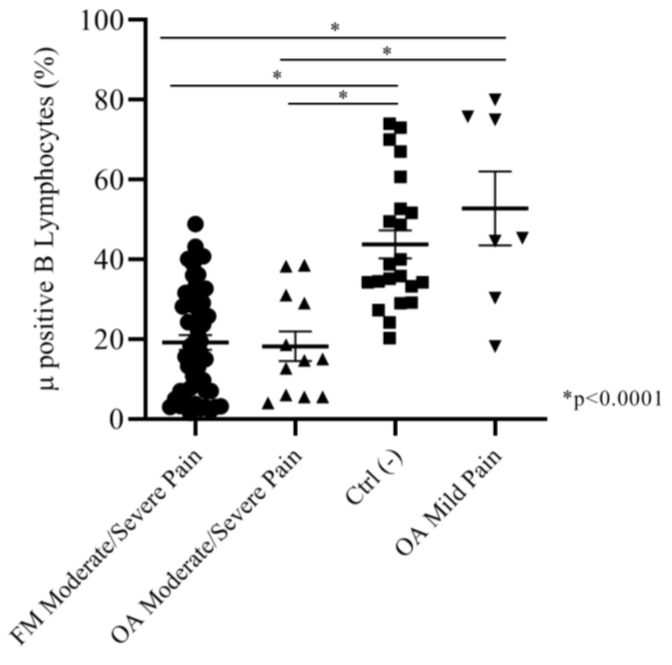
Moderate and severe pain (NRS) FM and OA patients did not show any significant difference in Mu+ B cells percentages. Both of the groups expressed Mu+ B cells percentages significatively lower than the negative control group and mild pain OA patients.

Considering that both moderate and severe pain FM and OA patients did not show any significant differences in Mu+ B cell expression, we assembled the subgroups together in order to compare Mu expression in patients with different pathologies but the same intensity of pain, versus the negative control group. We found that moderate/severe pain FM patients (mean: 19.19 ± 1.8) and moderate/severe pain OA patients (mean: 18.23 ± 3.7) had almost the same percentage of Mu+ B cells; both the groups were significantly different (*p* < 0.001) from the control group (mean: 43.80 ± 3.5) and the mild pain group patients (mean: 52.77 ± 9.2). These last groups did not show any significant differences in the percentage of Mu+ B lymphocytes (Figure 3).

#### 2.2.3. Correlation between Duration of Pain and Mu Positive B Cells

**Figure 4:**
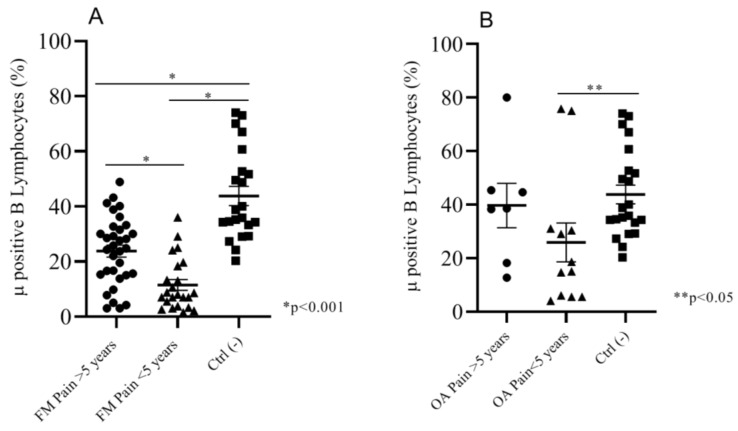
(**A**)FM patients suffering for more than 5 years showed a significantly higher percentage of Mu+ B cells than patients suffering for less than 5 years; the negative control group expressed a Mu+ B cells percentage that was significantly higher than both of the two FM subgroups. (**B**) OA patients suffering for less than 5 years showed a significantly lower percentage of Mu+ B lymphocytes than the negative control group.

We analyzed the percentage of expression of Mu+ B cells in both of the FM and OA subgroups versus the control group, by considering the number of years after pain insurgence. Fifty-six percent (56%) of FM patients (*n* = 33) were suffering for more than five years; the remainder were suffering for less than five years (54%, *n* = 26). We found a significant difference (*p* < 0.001) of percentages of Mu+ B cells between the two subgroups (respectively, mean: 23.80 ± 2.1; 11.50 ± 1.9) and between the single subgroups and the control pain-free group (mean: 43.80 ± 3.5) (Figure 4A). Sixty-three percent (63%) of OA patients (*n* = 12) were suffering for less than five years, and 37% (*n*=7) for more than five years. The percentage of Mu+ B cells was not significantly different between the two subgroups (respectively, mean: 39.67 ± 8.2; 25.88 ± 7.2). We found a significant difference (*p* < 0.05) in Mu+ B expression between the control group and OA patients suffering for less than five years (Figure 4B).

#### 2.2.4. Correlation between Drugs Therapy before Enrollment and Mu-Positive BCells

Both fibromyalgic and osteoarthritic patients require pharmacological therapies in order to alleviate their pain. In most cases, medical doctors also decide on a combination of drugs and prescribe formulations for psychological and psychiatric comorbidities, for FM patients. OA patients mostly used NSAIDs for inflammatory pain. Considering the pharmacological therapy at the time of enrolment, we analyzed whether Mu opioid receptor expression on B lymphocytes was influenced by the therapy. We considered the totality of patients and divided the entire number into the following therapy subgroups: antidepressants, benzodiazepines, NSAIDs, nutritional supplement/none, and previous drugs combination. We found that, despite the different pharmacological therapies, the percentage of Mu+ B cells was almost identical across the subgroups (mean: 18.3):antidepressant 17%; benzodiazepines 20%; NSAIDs 22%; nutritional supplements/none 21%; previous drugs combination 20%.

### 2.3. Correlations between Mu-Positive B Cells and Psychological Variables

Table 2 shows correlation coefficients among Mu+ B cells percentages and psychological variables in FM patients (*n* = 53). Mu+ B cell expression had no significant correlations with any psychological aspects investigated.

Given the similarities in Mu+ B cells percentages between FM and OA patients at the same intensity of pain (NRS (4–6), NRS (7–10)), we conducted the analysis considering together FM and OA patients with moderate and severe pain (*n* = 58). Table 3 Shows correlation coefficients among Mu+ B cells percentage and psychological variables. Mu+ B cells expression had no significant correlations with any psychological aspects investigated.

## 3. Discussion

Chronic pain diagnosis lacks specific tools to objectively define pathology, unlike other diseases (e.g., oncology or infectiology), where biomarkers play a well-known role. 

In this context, the objective evaluation of pain intensity is a crucial point, relevant for pharmacological management and especially for the choice of opioid therapy or to determine if or not to use a surgical approach (e.g., prosthesis implantation or neurostimulation). Thus, the proper diagnosis of pain severity has also an ethical and economical value, in terms of social and national health system costs.

The objectivity of pain severity is still described only in terms of pain threshold and patients have to rate their pain considering a numeric rating scale (NRS) of 0 to 10, where zero is a no pain condition and a 10 means the pain is as bad as it could be [50], even if it involves many other factors, such as anthropological, spiritual, genetic, social, and psychological personal experiences [51].

There is no internationally accepted classification of pain severity for FM or chronic diseases and the physician is tasked with careful evaluation in order to determine the most effective treatment [15]. It is often suggested that a distinction has to be made between mild, moderate and severe forms of FM, based on symptom intensity and functional disability [52].

Although FM has been included in the ICD-11 as “chronic primary pain” [7], a great number of clinicians still consider it as a somatoform pain disorder and FM diagnosis is based principally on the main symptom of widespread pain. As with other chronic pain syndromes, FM is characterized by non-specific and overlapping symptoms.

The absence of objective diagnostic tools is leading scientists to the identification of chronic pain markers, in order to improve the diagnostic process and reduce the risks of diagnostic delays and misdiagnosis [53,54]. An objective diagnosis could allow a better classification of these chronic diseases, by clarifying pathogenetic mechanism, prognosis and/or response to treatment, opening the way to personalized medicine. The importance of biomarkers to guide chronic pain diagnosis and treatment properly assumes an even greater impact, considering the lack of valid molecular markers for diagnosis for all the chronic pain states [53,55].

In this context, in recent years we have focused on the expression of the Mu opioid receptor on lymphocyte membrane, in order to identify this receptor as a hypothetical biomarker of chronic pain conditions. Focusing on the evidence that immune, nervous and opioid endogenous systems share the same opioid receptors, in the 1990sRaffaeli and colleagues studied and reported their crucial role in the pain pathways [56] and published a trial based on this idea related to osteoarthritic chronic pain in patients who underwent hip surgery [54].

In the present study, we certified that FM pain perception is an objective widespread chronic pain status where pain is the primary symptom and FM pathogenesis derives from a characteristic morphological modulation of the endogenous antinociceptive pathway. To demonstrate this, we chose OA as a chronic pain control group, considering our previous research on osteoarthritic pain [54] and its well-described nociceptive pain due to degenerative osteoarthritis status [57,58], and compared immunophenotype analysis of FM and OA patients with a pain-free negative control group.

We found that both FM and OA chronic pain patients present a diverse and significantly lower percentage of Mu+ B cells compared to the negative control group.

In our experiments, Mu receptor in T cells was expressed only by a very low frequency population (less than 2%) and there was no difference between the percentage of Mu+ T cells across the three groups, suggesting that the frequency of Mu+ T cells expression could not be a significant biological target in chronic pain patients. However, further investigation will be required to better understand this intriguing data.

Thus, we analyzed these data considering the intensity of the pain and found, for the first time, that FM or OA patients with moderate/severe intensity of pain on the NRS presented a lower percentage of B cells expressing Mu opioid receptor than patients in pain-free control group. Of interest is the finding that OA patients with a mild pain sensation showed a higher percentage of Mu+ B lymphocytes, which was not significantly different from the negative control group. 

The analysis of these data led us to postulate that FM patients effectively show a real pain condition, by having an overlapping opioid marker of the well-established OA patient pain, and their suffering is not merely psychosomatic expression.

As a clinical explanation for our results, we suggested that a lower expression of Mu+ B cells in FM and OA patients than in the negative control group seems to be linked to a reduced threshold level of pain response and that a low availability of opioid receptors in FM patients could show altered endogenous opioid analgesic activity. Similar results were presented about Mu opioid receptor accessibility in the central nervous system of FM patients, where a reduction was detected [59]. Thus, a scarce Mu opioid reserve could be an indicator of low cooperation of the endogenous opioid system in the prevention of chronic pain development. Understanding the role of Mu on lymphocytes could be helpful in the management of chronic pain-suffering patients undergoing rehabilitation programs. In the near future, it could be relevant to verify our findingin acute pain conditions and in inflammatory-related disease.

Raffaeli and coworkers here propose that this condition could be classified as the “B-Lymphocytic Reserve MOR Syndrome” theory. Even if further studies are needed in this direction, we postulate that during an endogenous or exogenous pain stimulus, the amount of B lymphocytes presenting MOR could contribute to counteract the algic response. Unlikely, when the intensity of pain appears more severe (high/moderate intensity) we assist to the reduction of Mu+ B cells percentage, and this deficit of B cells presenting MOR could affect pain perception and intensity, leading to an uncontrolled pain condition. Further studies are already ongoing to better identify the possible correlation of the different activation statuses of B lymphocytes with Mu+ Bcells frequency. In this study, we also found two different subpopulations of FM patients considering the duration of pain in years: here we show that FM patients suffering for more than five years have a lower percentage of Mu+ B cells than a subgroup of FM patients suffering for less than five years. In the near future, it could be interesting to analyze the modulation of the percentage of Mu+ B cells expression over the time, by following a specific group of patients from the first clinical evidence of FM.

Moreover, we found that the expression of Mu+ B cells is not associated with functional disability, illness representation, depression, anxiety, stress-related symptoms, coping, catastrophizing or pain acceptance. Therefore, these relevant psychological aspects do not seem to correlate with the biological findings, as shown in Table 2 and Table 3.

In conclusion, our results could contribute to the legitimization of FM as a real and serious chronic pain syndrome and could also be helpful in rethinking the description of pain categories: FM and OA are differently categorized in the ICD-11 but our results show similar biological characteristics. 

Due to this new evidence and to better depict the chronic pain scenario, new trials have already been submitted to the ethical committee in order to clarify whether the “B-Lymphocytic Reserve MOR Syndrome” hence the modulation of B cell presenting MOR is common to more than one chronic pain condition. Further investigation could help clinicians understand if these pathologies share the same pattern of pain.

## 4. Materials and Methods 

### 4.1. Trial Design

This is an observational, cross-sectional, single blind, diagnostic trial. The current study is approved by the institutional independent ethics committee of Sapienza University of Rome, with the name of “I markers Bio-Psico-Sociali nella syndrome fibromialgica” (Fibromyalgia syndrome Bio-Psycho-Social markers), on 8 March 2018 (Ref. 4937) and the trial is registered in on ISRCTN registry, ID: ISRCTN24645566, 10 December 2018.

### 4.2. Participants

All participants were enrolled at the Clinic for the Diagnosis and Therapy of Fibromyalgia, Rheumatology Unit, Sapienza University of Rome (Umberto I Policlinic).

Fifty-nine (59) consecutive adult patients affected by FM, according to both 1990 and 2010 ACR criteria [1,2], were enrolled. Inclusion criteria allowed the eligibility of adult (18–65 years old) FM patients, both males and females. 

Considering that opioid receptors and lymphocytes activity could be influenced by opioid treatment [19,20,21,22,23,24,25,26,27,28,29,30,31,32,33,34,35], patients currently being treated with opioids were not enrolled. However, information about the assumption of pharmacological therapy was collected. Exclusion criteria involve also patients with rheumatic pathologies.

A control group of 19OA patients (chronic pain control group) affected by chronic pain due to knee or hip degenerative osteopathy, ongoing active and/or passive therapy, paracetamol and NAISDs for mild/severe pain when needed, was involved.

A second control group was composed by 24 healthy people (pain-free negative control group).

All eligible and consenting patients signed a specific informed consent form and General Data Protection Regulation (GDPR) obligations, on the day of the first clinical visit. Each allocated patient, after inclusion/exclusion criteria analysis, clinical evaluation and consent form signature were included in a list, with a numeric sequential code, in order to protect confidentiality. Only the medical doctor could access patient names. All information was collected in electronic files. 

### 4.3. Clinical and Psychological Measurements

Clinical evaluation and socio-demographic characteristics were assessed by clinicians operating in Sapienza University.

Clinical data were collected during patients’ enrolment. Clinicians filled the database following a consequential numbering of the patients.

All patients and pain-free people underwent clinical examination. Questions were included about socio-demographic characteristics, duration of pain, comorbidities and current treatments. Clinical pain was reported on a 0–10 Numerical Rating Scale (NRS), with 0 for “no pain” and 10 for “the worst pain imaginable.”

A comprehensive battery of self-report tools in their Italian versions was administered to the patients of experimental and positive control groups. The Fibromyalgia Impact Questionnaire (FIQ; [60]) was administered in order to assess functional disability. The FIQ is a 10-item self-administered instrument that measures physical functioning, work status, depression, anxiety, sleep, pain, stiffness, fatigue and well-being. Higher scores indicate greater functional disability. In our study, the original FIQ was adapted in OA sample changing the terminology “fibromyalgia” into “osteoarthritis” (Osteoarthritis-FIQ Revised: OA-FIQ-R).

The Illness Perception Questionnaire-Revised (IPQ-R; [61]) is a 38-itemmeasure of illness representation in terms of perceived duration, consequences, understanding, control, and emotional impact of illness. Coping Strategies Questionnaire (CSQ; [62]) is a 27-itemmeasure of coping strategies used by patients to manage chronic pain, such as catastrophizing, distraction, denial, distancing, self-affirmation, and praying. Depression, Anxiety and Stress Scale-21 (DASS-21; [63]) is a 21-items measure of psychological distress in terms of anxiety, depressive and stress-related symptoms. Chronic Pain Acceptance Questionnaire (CPAQ; [64]) is a 20-itemmeasure of pain acceptance based on persistence in doing pleasant activities instead of trying to control pain and avoid activities. 

### 4.4. Blood Assays

Blood samples were collected during clinical examination. Blood samples were analyzed within the next 24 h through immunophenotyping analysis. The research biologists were blinded to patients’ personal information and therapy. They received patients’ samples in a sequential numeric code.

### 4.5. Biological Analysis

#### Immunophenotyping Analysis

Peripheral blood was incubated with fluorochrome-conjugated antibodies, specific for different cell population membrane markers, in combination with Mu anti-opioid receptor antibody. In particular, peripheral blood samples were stained with antibodies FITC conjugated anti CD3 (T lymphocytes), APC conjugated anti-Mu and PerCP-Cy5.5 conjugated anti-CD19 (B lymphocytes), all from Becton Dickinson, for 20 min at 4 °C. After staining, samples were incubated for 15 min at room temperature with BD FACS Lysing Solution (Becton Dickinson, Milano, Italy) and then centrifuged at 1500 RPM for five minutes. The resulting pellets were acquired immediately after adding PBS. Acquisition and analysis were performed on a LSR Fortessa X-20 flow cytometer (Becton Dickinson) using Diva software, in order to detect the percentage of Mu+ cells.

### 4.6. Statistical Analysis

Immunophenotyping results were analyzed by one-way ANOVA test for group comparisons and for intra-group homogeneity assessment, with Prism-GraphPad 8.0.2 software (San Diego, CA, USA). All data were expressed as mean ± S.E.M. Tukey’s least significant difference multiple comparison was used for post-hoc analysis following one-way ANOVA, to compute the probability values (P) in three-group comparison. A P threshold of 0.05 was considered statistically significant.

Descriptive statistics were used to describe socio-demographic data, diagnosis, pain intensity and pain duration.

Statistical analysis of psychological data was performed with SPSS 23 software. The Pearson’s parametric coefficient was used for the correlation analysis. 

## Figures and Tables

**Table 1:** Clinical characteristics: patients and healthy subjects were analyzed considering the diagnosis, the intensity of pain (11-point numerical rating scale (NRS)) and the duration of pain (years).

ClinicalCharacteristics	% Of Patients (*n*=102)	
**Diagnosis**		
Fibromyalgia (FM)	57.8 (*n* = 59)	
Musculoskeletaldisorders (OA)	18.6 (*n *= 19)	
Healthypeople	23.5 (*n *= 24)	
**Intensity of Pain (NRS)**	**FM **	**OA **
(1–3) MildPain	(*n *= 0)	42.1 (*n *= 8)
(4–6) Moderate Pain	15.3 (*n *= 9)	26.4 (*n *= 5)
(7–10) Severe Pain	84.7 (*n *= 50)	31.5 (*n *= 6)
None		
**Duration of Pain**	**FM**	**OA **
<5 years	59.3 (*n *= 35)	63.1 (*n *= 12)
>5 years	40.7 (*n *= 24)	36.9 (*n *= 7)

**Table 2:** Mu+ B cells percentage of expression had no significant correlations with any psychological aspects investigated. Pearson’s correlations between Mu+ B cells percentage of expression and total FIQ score, IPQ-R subscales, CSQ subscales, total CPAQ score and DASS subscales.

	Mu+ B Cells % of Expression
FIQ	−0.39
IPQ-R—Timeline acute/chronic	−0.101
IPQ-R—Timeline cyclical	−0.062
IPQ-R—Consequences	−0.093
IPQ-R—Personal control	0.056
IPQ-R—Treatment control	0.089
IPQ-R—Illnesscoherence	0.046
IPQ-R—Emotional representation	0.090
CSQ—Distraction	0.135
CSQ—Catastrophizing	−0.012
CSQ—Ignoring pain sensations	0.057
CSQ—Distancing from pain	0.107
CSQ—Coping self-statements	0.028
CSQ—Praying	−0.032
CPAQ	−0.021
DASS—Depression	0.064
DASS—Anxiety	0.053
DASS—Stress	0.105

Values are Pearson’s correlation coefficients.* *p* < 0.05; ** *p* < 0.01. FIQ = Fibromyalgia Impact Questionnaire; CSQ = Coping Strategies Questionnaire; CPAQ = Chronic Pain Acceptance Questionnaire; DASS-21 = Depression, Anxiety and Stress Scale.

**Table 3:** Mu+ B cells percentage of expression had no significant correlations with any psychological aspects investigated. Pearson’s correlations between Mu+ B cells percentage of expression and total FIQ score, IPQ-R subscales, CSQ subscales, the total CPAQ score and DASS) subscales.

	Mu+ B Cells % of Expression
FIQ/OA-FIQ-R	−0.027
IPQ-R—Timeline acute/chronic	−0.241
IPQ-R—Timeline cyclical	−0.067
IPQ-R—Consequences	−0.155
IPQ-R—Personal control	0.022
IPQ-R—Treatment control	0.122
IPQ-R—Illness coherence	0.006
IPQ-R—Emotional representation	0.133
CSQ—Distraction	0.055
CSQ—Catastrophizing	−0.014
CSQ—Ignoring pain sensations	−0.043
CSQ—Distancing from pain	0.036
CSQ—Coping self-statements	0.065
CSQ—Praying	0.024
CPAQ	0.031
DASS—Depression	−0.015
DASS—Anxiety	0.074
DASS—Stress	0.105

Values are Pearson’s correlation coefficients. * *p* < 0.05; ** *p* < 0.01. FIQ = Fibromyalgia Impact Questionnaire; OA-FIQ-R= Osteoarthritis- FIQ Revised; IPQ-R = Illness Perception Questionnaire-Revised; CSQ = Coping Strategies Questionnaire; CPAQ = Chronic Pain Acceptance Questionnaire; DASS-21 = Depression, Anxiety and Stress Scale.

## Abbreviations

FM	Fibromyalgia
ACR	American College of Rheumatology
OA	Osteoarthritis
ICD-11	International Classification of Diseases-11
CWP	Chronic Widespread Pain
PBMC	Peripheral Blood Mononuclear Cell
uHPLC-PDA-MS	Ultra-High Performance Liquid Chromatography–Photo Diode Array–Mass-Spectrometry
HPLC	High Performance Liquid Chromatography
MOR	Mu Opioid Receptor
DOR	Delta Opioid Receptor
KOR	Kappa Opioid Receptor
IgM	Immunoglobulin M
IgG	Immunoglobulin G
CRF	Case Report Form
NRS	Numerical Rating Scale
NSAIDs	Non Steroidal Anti InflammatoryDrugs
ANOVA	Analysis of Variance
CTRL	Control
FIQ	Fibromyalgia Impact Questionnaire
OA-FIQ-R	Osteoarthritis- FIQ Revised
IPQ-R	Illness Perception Questionnaire – Revised
CSQ	Coping Strategies Questionnaire
CPAQ	Chronic Pain Acceptance Questionnaire
DASS-21	Depression, Anxiety and Stress Scale-21
IRCCS	Istituto di Ricovero e Cura a Carattere Scientifico
GDPR	General Data Protection Regulation
